# Compound NSC84167 selectively targets NRF2-activated pancreatic cancer by inhibiting asparagine synthesis pathway

**DOI:** 10.1038/s41419-021-03970-8

**Published:** 2021-07-10

**Authors:** Bingbing Dai, Jithesh J. Augustine, Ya’an Kang, David Roife, Xinqun Li, Jenying Deng, Lin Tan, Leona A. Rusling, John N. Weinstein, Philip L. Lorenzi, Michael P. Kim, Jason B. Fleming

**Affiliations:** 1 Departments of Surgical Oncology, The University of Texas MD Anderson Cancer Center, Houston, TX 77030 USA; 2 Department of Gastrointestinal Oncology, H. Lee Moffitt Cancer Center and Research Institute, Tampa, FL 33612 USA; 3 Departments of Bioinformatics and Computational Biology, The University of Texas MD Anderson Cancer Center, Houston, TX 77030 USA

**Keywords:** Drug discovery, Gastrointestinal diseases

## Abstract

Nuclear factor erythroid 2-related factor 2 (NRF2) is aberrantly activated in about 93% of pancreatic cancers. Activated NRF2 regulates multiple downstream molecules involved in cancer cell metabolic reprogramming, translational control, and treatment resistance; however, targeting NRF2 for pancreatic cancer therapy remains largely unexplored. In this study, we used the online computational tool CellMiner^TM^ to explore the NCI-60 drug databases for compounds with anticancer activities correlating most closely with the mRNA expression of NQO1, a marker for NRF2 pathway activity. Among the >100,000 compounds analyzed, NSC84167, termed herein as NRF2 synthetic lethality compound-01 (NSLC01), was one of the top hits (*r* = 0.71, *P* < 0.001) and selected for functional characterization. NSLC01 selectively inhibited the viabilities of four out of seven conventional pancreatic cancer cell lines and induced dramatic apoptosis in the cells with high NRF2 activation. The selective anticancer activity of NSLC01 was further validated with a panel of nine low-passage pancreatic patient-derived cell lines, and a significant reverse correlation between log(IC_50_) of NSLC01 and NQO1 expression was confirmed (*r* = −0.5563, *P* = 0.024). Notably, screening of a panel of nine patient-derived xenografts (PDXs) revealed six PDXs with high NQO1/NRF2 activation, and NSLC01 dramatically inhibited the viabilities and induced apoptosis in ex vivo cultures of PDX tumors. Consistent with the ex vivo results, NSLC01 inhibited the tumor growth of two NRF2-activated PDX models in vivo (*P* < 0.01, *n* = 7–8) but had no effects on the NRF2-low counterpart. To characterize the mechanism of action, we employed a metabolomic isotope tracer assay that demonstrated that NSLC01-mediated inhibition of de novo synthesis of multiple amino acids, including asparagine and methionine. Importantly, we further found that NSLC01 suppresses the eEF2K/eEF2 translation elongation cascade and protein translation of asparagine synthetase. In summary, this study identified a novel compound that selectively targets protein translation and induces synthetic lethal effects in NRF2-activated pancreatic cancers.

## Introduction

Nuclear factor (erythroid-derived 2)-like 2 (NFE2L2), or nuclear factor erythroid 2-related factor 2 (NRF2), is a transcription factor that maintains cellular redox homeostasis by regulating the expression of antioxidant genes, including glutathione peroxidase, thioredoxin 1, thioredoxin reductase, peroxiredoxin 1, and sulfiredoxin 1 [1]. NRF2 also plays a critical role in cellular detoxification through enzymes such as NAD(P)H quinone oxidoreductase-1 (NQO1), glutathione S-transferases, and heme oxygenase-1 [1]. Under normal conditions, NRF2 is negatively regulated by Kelch-like ECH-associated protein 1 (KEAP1), which binds to NRF2 and the E3 ubiquitin ligase Cullin-3 (CUL3) protein to drive proteasome degradation of NRF2 [2–5]. Under oxidative or xenobiotic stress conditions, reactive cysteine groups of KEAP1 are modified, and NRF2 is released from the complex and translocated to the nucleus where it forms heterodimers with small musculoaponeurotic fibrosarcoma (sMAF) and promotes expression of a battery of genes involved in protection against oxidative damage, metabolism reprogramming, survival, proliferation, and other pathways [1, 6].

Mutations of *KEAP1*, *CUL3*, or *NRF2* can cause constitutive activation of NRF2 [7]. Besides gene mutations, other oncogenic signaling pathways, including AKT, MAPK, PKC, and c-Myc, also regulate KEAP1/NRF2 pathways through direct phosphorylation of NRF2 or through transcriptional or post-transcriptional regulation of NRF2 expression [7–9]. In addition, p21, PALB2, and p62 can also indirectly activate NRF2 through binding or phosphorylation of KEAP1 [10–13]. In pancreatic cancer, gene mutations in the KEAP1/NRF2 pathway are rare; however, NRF2 expression levels are high in over 93% of pancreatic adenocarcinomas [14]. The overexpression is believed to be a consequence of the near-universal presence of oncogenic *KRAS* gene mutations and downstream activation of the MAPK pathway and high levels of c-Myc [14, 15]. The oncogenic pathway-induced NRF2 expression in pancreatic cancer enhances ROS detoxification and modulates protein translation to promote tumorigenesis and drug resistance [14–16].

Several approaches have been explored to develop small-molecule drugs that target the NRF2 pathway [17]. Small-molecule inhibitors, including brusatol, ML358, K-563, and AEM1, which directly or indirectly target NRF2 or its downstream signaling, inhibit tumor growth and enhance the antitumor activity of chemotherapeutic agents in multiple cancer models [18–23]. Some repurposed agents, including clobetasol propionate, PHA-76749, PIK87, and dimethyl fumarate (BG-12), are not specific NRF2 inhibitors, but they can block NRF2 nuclear translocation or trigger degradation of NRF2 by targeting upstream signaling pathways [19, 24–26]. However, specific targeting of the NRF2 pathway for cancer therapy has yet to be achieved.

In this study, using a synthetic lethal approach, we identified a candidate compound NSC84167, termed as NRF2 synthetic lethal compound-01, NSLC01, which is selectively toxic to PDAC cancer cells and PDX tumors with high NRF2 activation. We found that NSLC01 specifically inhibited the protein translation of asparagine synthesis pathway in cells with high NRF2 activity. This compound might be worth future developments for the treatment of large subset of pancreatic cancers that show NRF2 activation.

## Materials and methods

### Reagents and animals

RPMI1640 and Dulbecco’s Modified Eagle Medium were purchased from HyClone. Fetal bovine serum and PrestoBlue Cell Viability reagent were purchased from Life Science Technologies (Grand Island, NY). Penicillin-streptomycin-neomycin (PSN) Antibiotic Mixture (100×) was purchased from Sigma (Missouri, MO). PARP, cleaved PARP, caspase-3, cleaved caspase-3, 4EBP (53H111), p-4EBP1 (S65), p-eEF2 (T36), p-eEF2K (S366), p-eIFA(S51), eEF2, NRF2, ATF4, and eEF2K were purchased from Cell Signaling Technology (Danvers, MA). Antibodies for D-3-phosphoglycerate dehydrogenase (PHGDH), phosphoserine phosphatase (PSPH), phosphohydroxythreonine aminotransferase 1 (PSAT1), and β-actin were purchased from Sigma. Asparagine synthetase (ASNS), NQO1, and heme oxygenase-1 (HO-1) antibodies were purchased from Santa Cruz Biotechnology. NSC84167 (oxo-7*H*-benzo[*e*]perimidine-4-carboxylic acid) was purchased from Sigma. Conventional pancreatic and non-small cell lung cancer cell lines were from American Type Culture Collection, and primary pancreatic cancer cells were established from PDXs in our laboratory as described previously [27]. Cell lines were authenticated by STR profiling in the Characterized Cell Line Core Facility at MD Anderson using established algorithms to confirm authenticity [28]. NOD/SCID and nude mice (female, aged 6 week) were purchased from Jackson Laboratories.

### Analysis of NCI-60 drug database with CellMiner^TM^

CellMiner™ (https://discover.nci.nih.gov/cellminer/) is a web application developed by the Genomics & Bioinformatics Group at National Cancer Institute (NCI) that facilitates systems biology through retrieval and integration of molecular and pharmacological data sets for the NCI-60 cell line panel [29, 30]. NQO1 mRNA expression was used as the marker of NRF2 activity to screen the NCI-60 cell line database with CellMiner^TM^, for chemical compounds whose activity is mostly correlated with NQO1 expression.

### MTT cell viability assay

Cell viability was measured with MTT assay as described previously [27]. The dose that causes 50% of cells death (IC_50_) of infected cells compared with non-infected control was calculated with GraphpadPrism software (8.0).

### Western blot analysis

Detailed methods can be found in our previous publications [27, 31].

### Flow cytometry

Cell cycle and apoptosis assays were performed as described previously [32].

### Measurement of ROS production

Cells were treated with NSLC01 for 24 h and then stained with 2′,7′-dichlorodihydrofluorescein diacetate (H2DCFDA) for 30 min following the manufacturer’s instructions. Cellular ROS levels were measured using a flow cytometer with a 488 nm laser for excitation and 535 nm laser for detection. The cell population with fluorescence was analyzed using Kaluza Analysis Software (V2, Beckman).

### Luciferase reporter assay

NRF2 transcriptional activity was analyzed using luciferase reporter as described previously [32]. ARE-Luc plasmid was received as a gift from Dr. Donna Zhang, Arizona University, Tucson, AZ.

### Real-time polymerase chain reaction (PCR)

Real-time PCR primers for human GHPDH, PSAT1, PSPH, and ASNS were purchased from Bio-Rad. Reactions were run using iQ SYBR Green Supermix on a CFX96 PCR machine (Bio-Rad). Gene expression was calculated using the 2^−ΔΔCT^ method.

### Immunohistochemistry (IHC) staining of paraffin-embedded tumor tissues

Immunohistochemistry was performed as described in a previous publication [33]. Images were captured with an Olympus DP72 camera and CellSens software (Center Valley, PA) on an Olympus BX51 microscope.

### Establishment of PDXs

Pancreatic cancer xenografts were established from patients with written informed consent for our institutional review board-approved laboratory research protocol (LAB07-0854). Briefly, fresh tumor samples were cut into fragments of about 2–3 mm [3], briefly soaked in Matrigel, and implanted in the subcutaneous space of NOD/SCID mice as described previously [33, 34].

### Live tissue sensitivity assay (LTSA)

LTSA was performed as described previously [33]. Tissue slices were treated with NSC84167 at different doses for 72 h. Tissue slice viabilities were measured using PrestoBlue reagent by reading the fluorescence intensity with a CLARIOstar plate reader (BMG LABTECH, Cary, NC), and these readings were normalized against the viability of untreated control slices.

### In vivo experiments

The animal experimental protocol was reviewed and approved by The University of Texas MD Anderson Cancer Center (Houston, TX) institutional review board, in accordance with the Guidelines for the Care and Use of Laboratory Animals. Power analysis was used to determine the animal number in each group. With each PATX tumor fragment was mixed with an equal volume of Matrigel (Invitrogen, Carlsbad, CA) and implanted into a nude mouse. When tumors reached ~100 mm^3^, mice were randomly divided (simple randomization method) into 2 groups of 5–10 mice and treated by intraperitoneal injection with vehicle (10% Cremophor EL, 12.5% polyethylene glycol 400, and 15% ethanol) or vehicle formulated NSLC01 (50 mg/kg). Investigator was blinded to the group allocation when tumors were measured weekly. Tumor volumes were calculated using the formula length × width^2^ × 0.52. When tumors reached ~15 mm in diameter, the mice were euthanized, and the tumors were harvested for histochemical analysis.

### Analysis of amino acid metabolites by liquid chromatography/high-resolution mass spectrometry

Incorporation of glucose carbon (^13^C_6_-glucose) and glutamine nitrogen (^15^N_2_-glutamine) into amino acids were analyzed using ultrahigh-resolution mass spectrometry. Cells were seeded in 10 cm dishes and grown to approximately 80% confluency. The cells were treated with NSLC01 in 0.1% DMSO or control (0.1% DMSO) for 24 h, and the medium was then replaced with fresh RPMI medium containing 5% FBS with 10 mM 13C6-glucose, 2 mM 15N2-glutamine, and 3 µM NSLC01 in 0.1% DMSO or control (0.1% DMSO) for an additional 24 h. Cells were washed twice with isotonic saline solution (0.9% NaCl), and metabolites were extracted using cold 80/20 (v/v) acetonitrile/water. For amino acid analysis, evaporated samples were reconstituted in 7.5 µL water containing 0.1% formic acid, followed by 42.5 µL acetonitrile containing 0.1% formic acid. Then, 15 μL portions of the solution were injected into a Thermo Vanquish liquid chromatography system containing an Imtakt Intrada Amino Acid column (2.1 × 150 mm) with 3 µm particle size. Mobile phase A was acetonitrile containing 0.1% formic acid. Mobile phase B (MPB) was 50 mM ammonium formate. The flow rate was 300 µL/min (at 35 °C), and the gradient conditions were as follows: initial 15% MPB, increased to 30% MPB at 20 min, then increased to 95% MPB at 30 min, held at 95% MPB for 10 min, returned to initial conditions and equilibrated for 10 min. The total run time was 50 min. Data were acquired using a Thermo Orbitrap Fusion Tribrid mass spectrometer under the ESI-positive ionization mode at a resolution of 240,000. The raw files were imported to Thermo Trace Finder software for final analysis. The fractional abundance of each isotopolog was calculated using the peak area of the corresponding isotopolog divided by the sum of all isotopolog areas.

### Protein translation assay

The effect of NSLC01 on synthesis was analyzed using the Click-It HPG Alexa Fluor 549 kit (Thermo Fisher). Cells were treated in four-well chamber slides with DMSO or NSLC01 (3 µM) for 24 h, and then a protein synthesis assay was performed following the manufacturer’s instruction. Images were captured with an Olympus DP72 camera and CellSens software (Center Valley, PA) on an Olympus BX51 microscope.

### Statistical analysis

Significant treatment-group differences were tested using one-way analysis of variance or *t* tests (two-tailed), with *P* < 0.05 considered statistically significant. Correlation of NQO1 expression with sensitivity of cell lines to NSLC01 was analyzed using Pearson correlation. All statistical analyses were done with GraphPad Prism 7.0 (GraphPad Software Inc., La Jolla, CA).

## Results

### Identification of the NSC84167 compound (NSLC01) as synthetically lethal with high NRF2 activation

**Fig. 1:**
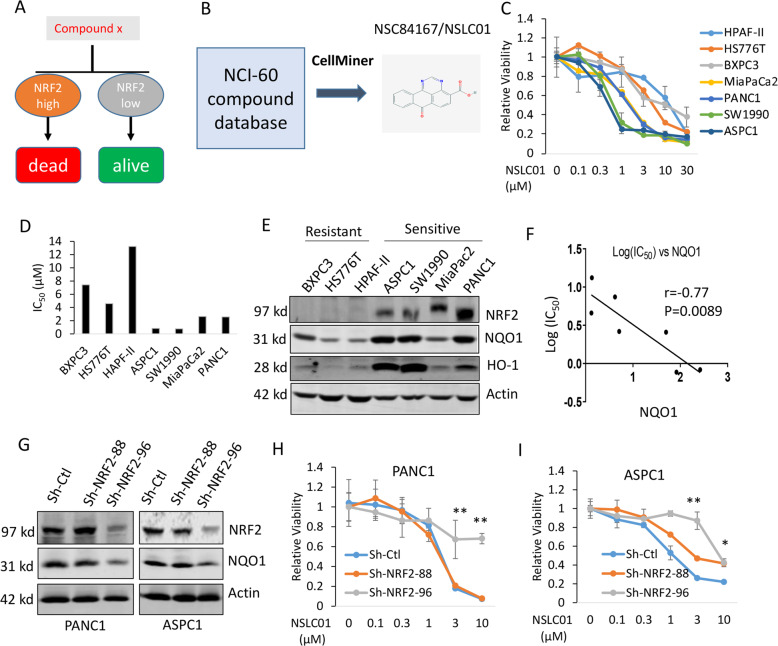
Identification of the NSC84167 compound (NSLC01) as synthetically lethal with high NRF2 activation. **A** Schematic illustration of the synthetic lethal strategy of targeting cancer cells with high NRF2 activity. **B** Working flow of identification of NSC84167/NSLC01. **C** Response curves of seven pancreatic cancer cell lines to NSLC01. Data are presented as mean ± SD, *n* = 3. **D** IC_50_ values of NSLC01 in the seven cell lines. **E** Western blot analysis of NRF2 and NQO1 expression in pancreatic cancer cell lines. **F** Correlation of NQO1 expression with the IC_50_ of NSLC01 in the seven cell lines. **G** Western blot analysis of the knockdown of NRF2 in PANC1 and ASPC1 cell lines. **H** Response of PANC1 cells to NSLC01 with NRF2 knockdown (^**^P < 0.01). **I** Response of ASPC1 cells to NSLC01 with NRF2 knockdown (^*^P < 0.05; ^**^P < 0.01). Data in (**H**) and (**I**) are mean ± SD, *n* = 3.

The goal of this study was to identify compounds that are selectively toxic to pancreatic cancer cells with high NRF2 activity through a synthetic lethal approach (Fig. 1A). NQO1 is a detoxification gene that is regulated by the NRF2 pathway, and NQO1 gene expression or promoter activity has been used as a reporter for NRF2 activity [2, 35]. Using NQO1 mRNA expression as the marker of NRF2 activity and CellMiner^TM^ (https://discover.nci.nih.gov/cellminer/), an online database tool, we analyzed the NCI-60 cell line database for chemical compounds whose activity is correlated with NQO1 expression. Our analysis identified NSC84167 (7-oxo-7*H*-benzo[*e*]perimidine-4-carboxylic acid) as one of the top hits; its pattern of growth inhibition across the 60 cell lines correlated most closely with NQO1 expression (*r* = 0.71, *P* < 0.001) (Fig. 1B and Supplementary Table 1). In addition, we also confirmed that NSC84167 is the top hit when HO-1, another NRF2-regualted molecule, was used for the same analysis using NCI-60 cell line database (Supplementary Table 2). We termed this compound as NRF2 synthetic lethal compound-01 (NSLC01) for better description.

To test the anticancer activity of NSLC01 against pancreatic cancer cells in vitro, we performed MTT cell viability assays with a panel of seven conventional pancreatic cancer cell lines. Four out of the seven, PANC1, MiaPaCa2, SW1990, and ASPC1, were sensitive to NSLC01 (IC_50_ of 3 µM as an arbitrary cutoff), whereas HPAF-II, HS776T, and BXPC3 were resistant (Fig. 1C, D). Immunoblotting confirmed that NRF2, NQO1, and HO-1 expression levels were higher in sensitive cells than in resistant cells (Fig. 1E). Correlation analysis indicated a strong negative correlation between NQO1 expression and log(IC_50_) (*r* = −0.77, *P* = 0.0089; Fig. 1F). In addition, we also assessed the activity of NSLC01, an immortalized normal pancreatic ductal epithelial cell line (hTERT-HPNE) and non-small cell lung cancer cells with or without constitutive NRF2 activation due to different mutation statuses of *KEAP1* gene [36]. The results showed that two *KEAP1* mutant cell lines (H838 and H460) were very sensitive to NSLC01, whereas, two *KEAP1* wild-type counterparts (HCC193 and H1299) and hTERT-HPNE were resistant to NSCLC-01 (Supplementary Fig. 1). These results indicate that NSLC01 is also active in NRF2-activated cancer cells due to genetic mutations of the components in KEAP1/NRF2 pathway but not in *KEAP1* gene wild-type or immortalized normal human pancreatic ductal epithelial cells.

To further test whether activation of the NRF2 pathway is causally involved in the growth inhibition by NSC84167, we used shRNA to knock down NRF2 in the PANC1 and ASPC1 cell lines and performed cell viability assays. The results showed that knockdown of NRF2 with sh-NRF2–96 inhibited NQO1 expression in PANC1 and ASPC1 cells and reversed NSC84167-induced growth inhibition in both PANC1 and ASPC1 cells, whereas shRNA-control (sh-Ctl) or sh-NRF2–88, which has no knockdown effect, did not affect the activity of NSC84167 (Fig. 1G–I).

Taken together, these results indicate that the anticancer activity of NSLC01 is correlated with activation of NRF2.

### NSLC01 induced apoptosis in NRF2-activated pancreatic cancer cells independent of ROS

**Fig. 2:**
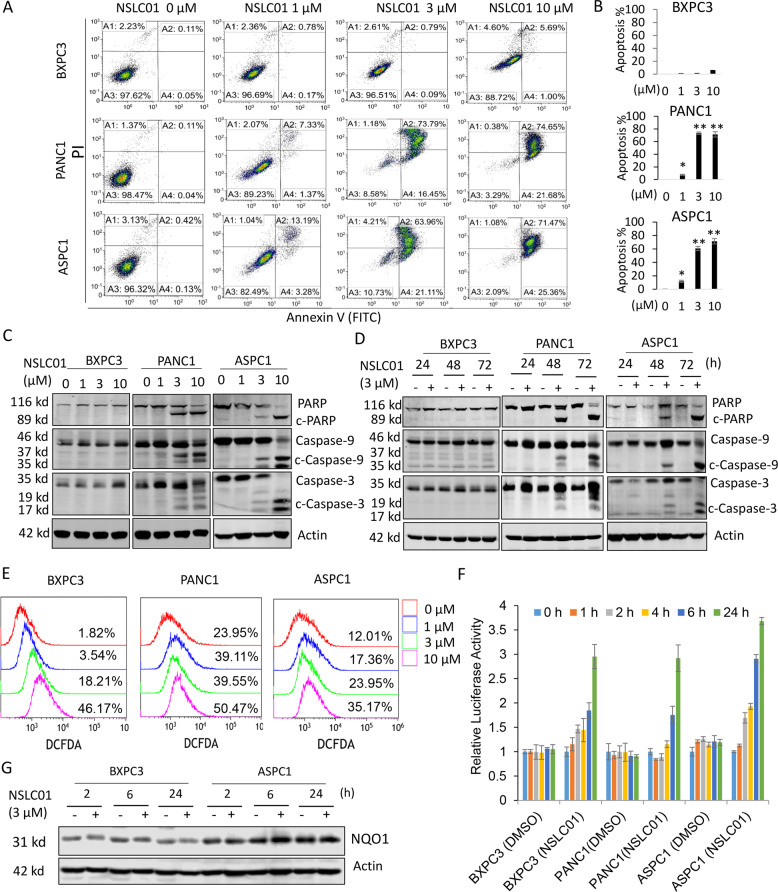
NSLC01 induces apoptosis in pancreatic cancer cells. Cells were treated with various doses of NSLC01 for 48 h and then stained with propidium iodide (PI) and Annexin V FITC for flow cytometry analysis of apoptosis. **A** Representative results from triplicate experiments. **B** Quantitation of apoptosis populations. Data are presented as mean ± SD; *n* = 3; ^*^P < 0.05; ^**^P < 0.01. **C** Western blot analysis at 48 h to measure apoptosis markers, cleave- (c-) PARP, c-caspase-3, and c-Caspase-9. **D** Western blot analysis to measure c-PARP, c-caspase-3, and c-caspase-9 in cells treated with 3 µM NSLC01 for various durations. **E** ROS measurements after treatment with various doses of NSLC01 for 24 h. **F** Cells were transfected with 1 µg of ARE-Luc promoter plasmids and 0.1 µg of actin-RLuc internal control plasmids, and after 24 h, cells were treated with NSLC01 at indicated time periods and ARE-Luc promoter reporter activity were measured. Data are presented as mean ± SD. There were no statistically significant differences between the cell types. **G** Western blotting analysis of NQO1 expression in BXPC3 and ASPC1 after treatment with NSLC01 for indicated time.

To further investigate the mechanism of action of NSLC01, we performed an apoptosis assay using flow cytometry. Exposure of cells to 3 or 10 µM NSLC01 for 48 h induced dramatic apoptosis in sensitive lines but no evident effect in the resistant line (Fig. 2A, B). Western blotting confirmed that 3 or 10 µM NSLC01 induced a dose-dependent effect in sensitive lines, PANC1 and ASPC1, as measured by cleavages of PARP, caspases-9, and caspases-3 (Fig. 2C). Of note, these effects in sensitive cells were strongest after 48 or 72 h of treatment and less so after only 24 h (Fig. 2D). Results from previous studies have indicated that apoptosis observed in targeting the NRF2 pathway is a consequence of ROS production [37]; however, NSLC01 induced limited ROS in both sensitive and resistant pancreatic cancer cell lines (Fig. 2E). In addition, NSLC01 did not inhibit the transcriptional activity of NRF2 or downstream NQO1 expression in either sensitive or resistant cell lines (Fig. 2F, G). These results indicate that NSLC01 selectively induces apoptosis of pancreatic cancer cells with high NRF2 activation, and the effect appears to be independent of NRF2 transcriptional activity or ROS production.

### NSLC01 selectively inhibited the viabilities of patient-derived pancreatic cancer cells and ex vivo-cultured PDX tumor tissues

**Fig. 3:**
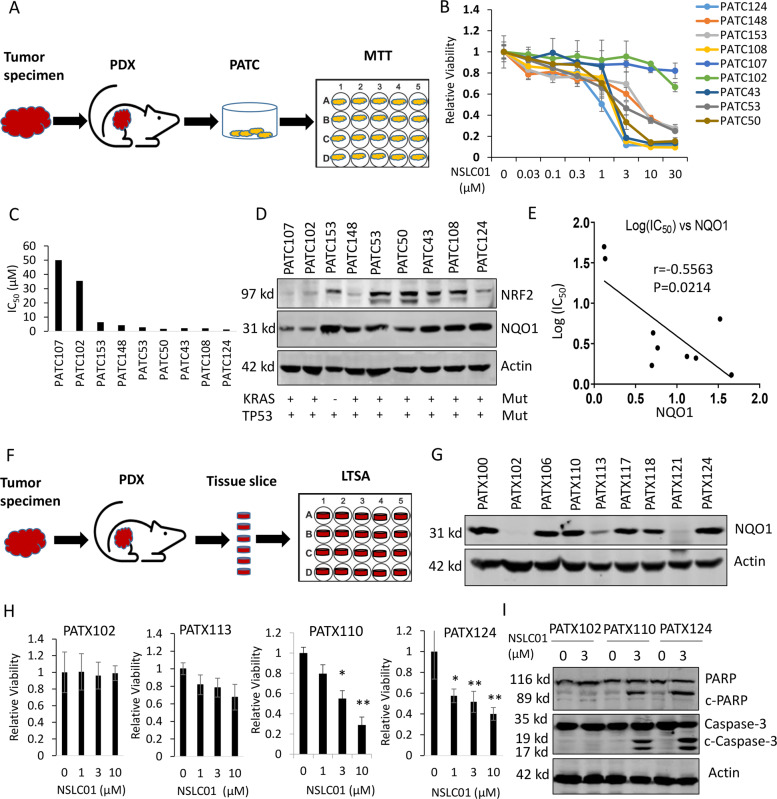
NSLC01 inhibited the viabilities of patient-derived pancreatic cancer cells and ex vivo-cultured PDX tumor tissues. **A** Schematic illustration of establishing patient-derived pancreatic cancer cells from PDX tumors for drug activity testing. **B** Response profile of patient-derived pancreatic cancer cells to NSLC01. **C** IC_50_ of NSLC01 in nine PDX-derived pancreatic cancer cell lines. **D** Western blot analysis of the expression of NRF2 and NQO1 in patient-derived pancreatic cancer cells and the mutation status for *KRAS* and *TP53* genes. **E** Correlation between NQO1 expression and the IC_50_ values of NSLC01 in patient-derived pancreatic cancer cells. **F** Schematic illustration of ex vivo organotypic tissue slice culture and LTSA assay with PDAC PDX tumors. **G** Western blot analysis of NQO1 expression in pancreatic cancer PDXs. **H** Ex vivo testing of NSCL01 activity with four selected PDAC PDX models using LTSA assay. Tissue slices were generated from PDX tumors as described in method section and deployed in 96-well plate. Tissue slices were treated with indicated concentrations of NSLC01 for 72 h, then tissue slice viabilities were measured with PrestoBlue^®^ reagent. ^*^P < 0.05; ^**^P < 0.01. Data are presented as mean ± SD, *n* = 3. **I** Western blot analysis of two apoptosis markers, cleaved PARP and cleaved caspase-3, in PDXs after treatment with NSLC01.

To further validate the anticancer activity of NSLC01 and its correlation with NRF2/NQO1 pathway activation, NSLC01 was tested in a panel of nine low-passage (less than 10 passages) patient-derived pancreatic cancer cell lines established from our PDAC PDXs (Fig. 3A.). In MTT cell viability assays, four out of the nine patient-derived cell types, PATC124, PATC108, PATC43, and PATC50, were sensitive to NSLC01; PATC53, PATC148, and PATC153 were moderately sensitive, and PATC102 and PATC107 were most resistant (Fig. 3B, C). Western blotting showed that, overall, NRF2 and NQO1 expression levels were higher in sensitive cells than that in resistant cells (Fig. 3D). Pearson correlation analysis further confirmed the inverse correlation between NQO1 expression and log (IC_50_) of NSLC01 (*r* = 0.5563, *P* = 0.0214; Fig. 3E), suggesting that NRF2 pathway activation is associated with the response to NSLC01. Whole-exome sequencing further revealed that eight out nine PATC lines harbor both *KRAS* and *TP53* mutations, whereas PATC153 has a wild-type *KRAS* gene (Fig. 3D and Supplementary Table 3). Mutation statuses of other selected genes including *BRCA1*, *BRACA2*, and *PALB2* are listed in Supplementary Table 3, though no association was found between specific gene mutations and NSCL01 sensitivity.

The selective anticancer activity of NSLC01 was further tested using a previously established LTSA method [33], an ex vivo drug sensitivity testing with organotypic culture of tissue slices generated from PDAC PDXs (Fig. 3F). A panel of nine pancreatic PDXs was randomly selected from our PDXs bank and screened by western blotting for NQO1 expression. Three PDXs, PATX102, PATX113, and PATX121, showed low or non-detectable expression of NQO1, while six PDXs showed high NQO1 expression (Fig. 3G). Notably, PATC102 cell line, which was derived from the PDX models of PATX102, also showed low expression of NQO1 and was resistant to NSLC01 (Fig.  3B,D). Using the ex vivo LTSA assay (Fig. 3F), we confirmed that PDX tumors with high NQO1 expression (PATX110 and PATX124) were sensitive to NSLC01, whereas PDXs with low NQO1 expression (PATX102 and PATX113) were resistant (Fig. 3H). In addition, NSLC01 induced cleavage of PARP and caspase-3 in NQO1-high, but not in NQO1-low, PDXs (Fig. 3I). Taken together, these results demonstrate that NSLC01 is active against clinically relevant pancreatic cancer cell lines and ex vivo-cultured tumor tissues, with cytotoxic effects based on NRF2/NQO1 activation.

### NSLC01 inhibited tumor growths of NRF2-activated PDAC PDX models in vivo

**Fig. 4:**
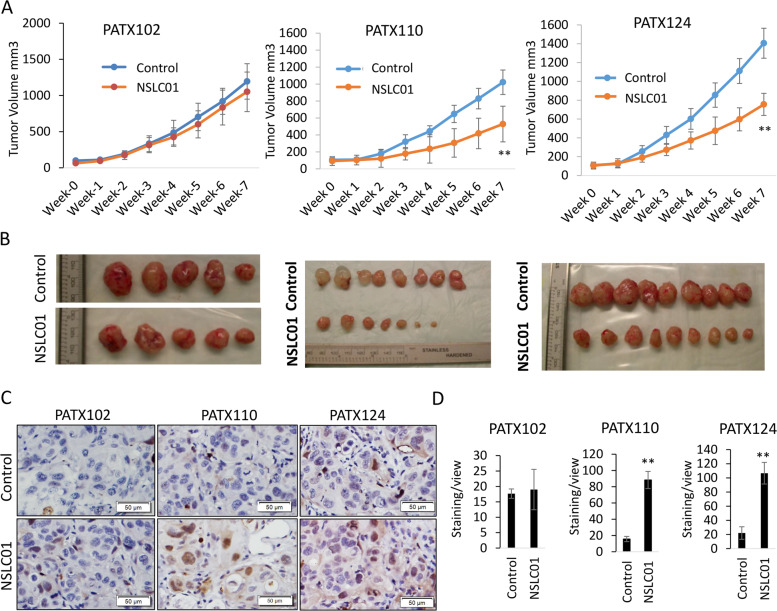
NSLC01 inhibited tumor growths of NRF2-activated PDAC PDX models in vivo. **A** Tumor growth of PATX102 (resistant), PATX110 (sensitive), and PATX124 (sensitive). PDXs models were established in nude mice, and when tumors reached the sizes of ~100 mm^3^, mice (5–8 mice per group) were treated with vehicle control or NSCL01 (50 mg/kg) for 6 weeks. **B** Gross tumors from control and NSCL01-treated groups. **C** Immunohistochemistry staining of cleaved caspase-3 in tumor specimens after treatment. **D** Quantitation of cleaved capase-3 in immunohistochemistry analysis. ^**^P < 0.01.

Based on the ex vivo LTSA assay, we chose two NSLC01-sensitive PDXs (PATX110 and PATX124) and one resistant PDX (PATX102) to further validate the anticancer activity of NSLC01 in vivo. Compared with vehicle, NSLC01 significantly inhibited tumor growth of two sensitive PDXs, PATX110 (*p* < 0.01) and PATX124 (*p* < 0.01). These results were consistent with the ex vivo results (Fig. 4A, B). Animals treated with NSLC01 did not demonstrate any obvious toxicity based on body weight change or behavior (data not shown). Immunohistochemistry indicated that NSLC01 induces significant cleaved caspase-3 in NSLC01-sensitive, but not in resistant, PDXs (Fig. 4C, D), suggesting that NSLC01 also induced cancer cell apoptosis in vivo.

### NSLC01 inhibited asparagine de novo synthesis pathway by targeting protein translation

**Fig. 5:**
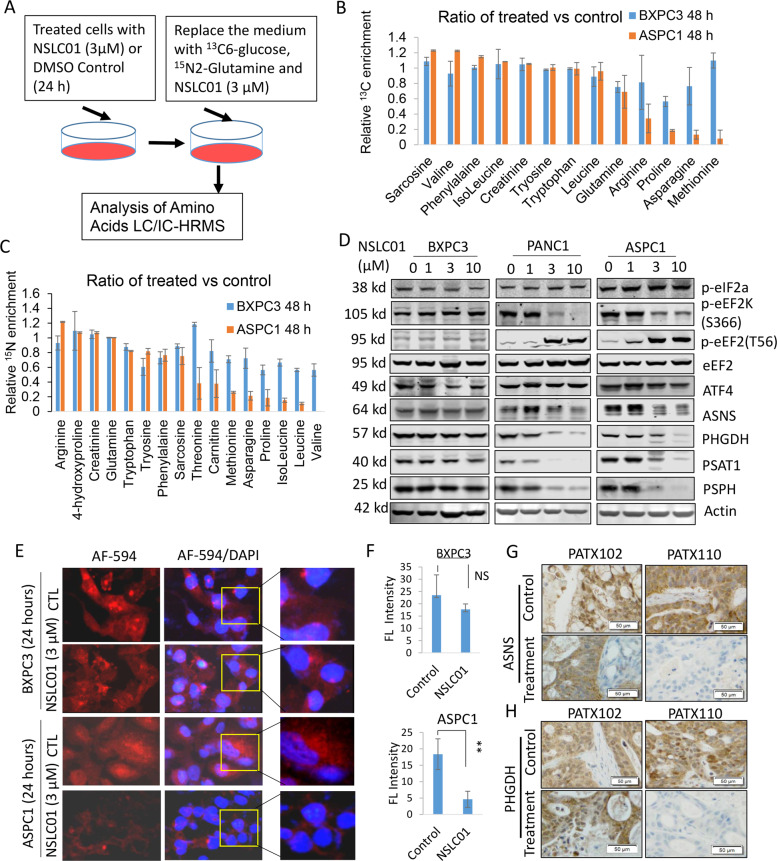
NSLC01 inhibits the asparagine de novo synthesis pathway by targeting protein translation. **A** Experimental flow for the metabolic flux assay: BXPC3 (resistant to NSLC01) and ASPC1 (sensitive to NSLC01) were treated with NSC or DMSO for 24 h; cell medium was replaced with fresh medium supplemented with ^13^C-glucose, ^15^N2-glutamine, and NSLC01 (3 µM) for an additional 24 h; and analysis of amino acids was performed using liquid chromatography/high-resolution mass spectrometry. **B**, **C** The relative ^13^C (**B**) and ^15^N (**C**) enrichment ratios of metabolites with detectable ^13^C or ^15^N enrichment were calculated using the percentage of ^13^C-labeled or ^15^N-labeled metabolite in NSLC01-treated cells compared with those in control-treated cells. Metabolites without detectable ^13^C or ^15^N enrichment are not shown. **D** Western blot analysis of the key molecules in protein translation and, asparagine and serine synthesis pathways. **E** Protein synthesis assay using Click-It HPG kits: cells were treated with NSLC01 (3 µM) or control for 48 h, and nascent protein synthesis was analyzed with Click-It HPG Alexa Fluor protein synthesis assay kits. Red color indicates nascent synthesized proteins. **F** Quantifications of fluorescence inensity. **G**, **H** Immunohistochemistry staining of asparagine ASNS and PHGDH, respectively, in tumor specimens after treatment.

Previous studies have shown that activation of the NRF2 pathway can reprogram cancer cell metabolism and upregulate multiple genes involved in the synthesis of serine, glycine, and asparagine, fueling cancer cell growth and survival [38–40]. To explore the inhibition of those processes as a possible mechanism of action of NSLC01, we used metabolomic tracer assay, with ^13^C-glucose and^15^N2-glutamine as tracers, to analyze the metabolites involved in de novo synthesis of amino acids (Fig. 5A). NSLC01 dramatically inhibited de novo synthesis of asparagine, proline, and methionine based on the ^13^C enrichment, by 80–90% in ASPC1, a sensitive cell line, and ~30% in BXPC1, a resistant counterpart (Fig. 5B). Whereas, from the ^15^N enrichment analysis, valine, methionine, leucine, isoleucine, proline, and asparagine were dramatically inhibited in sensitive cells but not in NSCLC01-resistant cells (Fig. 5C). In addition, we found that addition of exogenous asparagine in cell culture medium, to certain extent, rescued the cell growth inhibition mediated by NSCL01 treatment (Supplementary Fig. 3). These results suggest that inhibiting biosynthesis of these amino acids is involved in the anticancer effect of NSLC01.

It has been reported that NRF2 regulates the key enzymes involved in serine and asparagine synthesis pathways, including PHGDH, PSAT1, PHSH, and ASNS [39, 40]; however, in this study, we did not observe much effect of NSLC01 on the mRNA transcription of PHGDH, PSAT1, or ASNS (Supplementary Fig. 2). In contrast, protein expression of ASNS, PHDGH, PSAT1, and PSPH was dramatically reduced in sensitive cell lines (PANC1 and ASPC1), but not in resistant cell line (BXPC3), after treatment with NSLC01 (Fig. 5D). These results suggested that NSLC01 affects protein translation in sensitive cells. We then surveyed the phosphorylation levels of several enzymes involved in protein translation initiation and elongation and found that NSLC01 decreased phosphorylation of eukaryotic elongation factor 2 kinase [p-eEF2K (S366)], while increased phosphorylation of eukaryotic elongation factor 2 [p-eEF2(T56); (Fig. 5D)]. Phosphorylation of eEF2K at S366 inhibits its activation, suggesting that NSLC01 can activate eEF2K by reducing the inhibitory phosphorylation of S366. Activated eEF2K phosphorylates downstream eEF2 at T56, an inhibitory phosphorylation site, thereby inhibiting its function in the protein translation elongation process. In contrast, NSLC01 has no effect on translation initiation factor eIF2-alpha (eIF2a), suggesting no effect on the translation initiation step. In addition, ATF4, a downstream co-transcription factor of NRF2 in regulating ASNS, PHGDH, and PSAT1, was not inhibited by NSLC01 (Fig. 5D), which also confirms that NSLC01 acts to alter expression through protein translation but not transcription.

To further validate the inhibition of protein translation by NSLC01, we analyzed protein biosynthesis using protein synthesis kit Click-It HPG Alexa Fluor 549 (ThermoFisher) which checks protein synthesis by fluorescence imaging without using isotopes. NSLC01 dramatically inhibited protein translation in sensitive ASPC1 cells but not in resistant BXPC3 cells (Fig. 5E). In addition, immunohistochemistry also confirmed that the expression of both ASNS and PHGDH was reduced by NSLC01 in sensitive PATX110, whereas no inhibition of ASNS or PHGDH was observed in the resistant PATX102 (Fig. 5G, F). These results indicate that NSLC01 reduced the expression of asparagine and serine synthesis enzymes in PDX tumors with high NRF2 activity.

**Fig. 6:**
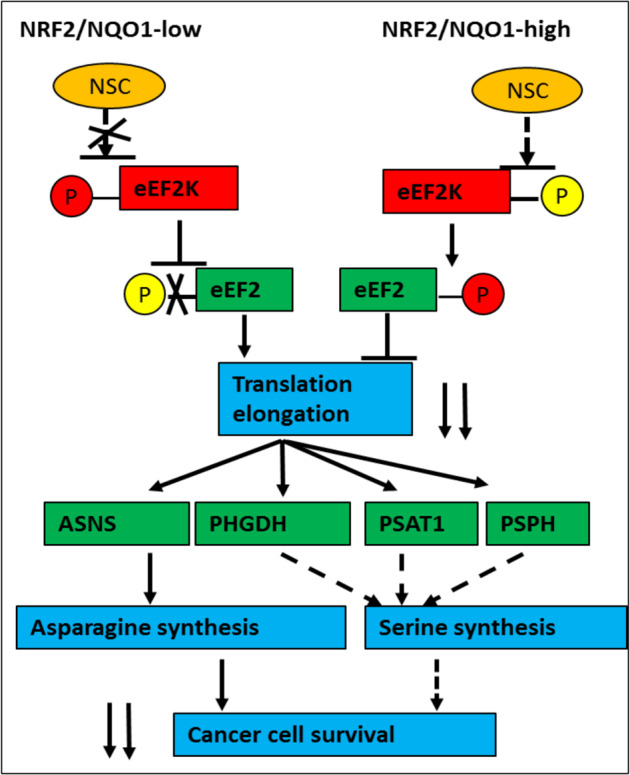
Proposed model of selective anticancer activity of NSLC01 in cancer cells with high NRF2 activation. NSLC01 selectively activates eEF2K by inhibiting its phosphorylation at S366; activated eEF2K then phosphorylates the downstream eEF2 at T56, inhibiting its translation elongation function; this inhibition of translation reduces the availability of enzymes (ASNS, GHPDH, PSAT1, and PSPH) that drive the synthesis of asparagine and likely, serine as well necessary for cancer cell survival and proliferation.

On the basis of these data, we propose the following as the mechanism of action model for NSLC01 (Fig. 6): NSLC01 selectively activates eEF2K by inhibiting its phosphorylation at S366; activated eEF2K then phosphorylates the downstream eEF2 at T56, thus inhibiting its translation elongation function; this inhibition of translation reduces the availability of enzymes (ASNS, GHPDH, PSAT1, and PSPH) that drive the synthesis of asparagine, and likely, serine, necessary for cancer cell survival and proliferation.

## Discussion

Synthetic lethal strategies have been actively explored in order to target cancers with dysregulated pathways or specific gene mutations. Several therapeutics developed based on synthetic lethality approaches have been successful in the clinic. For example, PARP inhibitors are selectively effective in BRCA mutant cancers [41]. In our study, the anticancer activity of NSLC01 closely correlated with NQO1 gene expression, a marker for NRF2 activation, and knockdown of NRF2 partially reversed the inhibitory effects of NSLC01, suggesting a linkage between activation of NRF2 and sensitivity to NSLC01. Previous study revealed that *KRAS* mutation upregulates NRF2 expression through activation of MAPK pathway, and indeed, we observed that BXPC3 cell line harboring a wild-type *KRAS* gene is resistant to NSLC01 (Fig. 1E), however, *KRAS* mutation alone cannot predict NSLC01 activity (Figs. 1E and 3D), since multiple mechanisms are involved in regulating NRF2 expression and activity. Mechanistically, it is possible that NSLC01 is activated by NQO1 which has been demonstrated to be a bio-activator of beta-lapachone [42]. Nevertheless, the biological functions of NSLC01 and its derivatives remain largely unknown. There was one early report showing that this compound and its derivatives might be antagonists of corticotropin-releasing factor (CRF) receptor [43], but it is unlikely that blockade of the CRF receptor by NSC84167 is involved in its selective anticancer activity in pancreatic cancer, since ductal pancreatic cancers lack the CRF receptor [44].

Previous studies have shown that NRF2 regulates the de novo synthesis of serine through regulating PHGDH, PSAT1, and PHSH in non-small cell lung cancer cells [39], and from the western blotting we also observed that NSLC01 inhibited the expression of these three molecules; however, in our tracer experiments, we did not detect any ^13^C or ^15^N enrichment in either serine or glycine in cells with or without NSLC01 treatment, whereas ^13^C or ^15^N enrichment could be detected in most other amino acids examined, suggesting that the de novo synthesis activity of serine and glycine are low in the tested pancreatic cancer cells. Our results showed that the de novo synthesis of asparagine and methionine were dramatically inhibited by NSLC01 in sensitive but not in resistant cells. Also, we found that addition of exogenous asparagine in cell culture medium, to some extent, rescued the cell growth inhibition mediated by NSCL01 treatment (Supplementary Fig. 3), indicating that inhibition of asparagine at least partially involved NSLC01-mediated anticancer activity. It has also been reported that intracellular asparagine regulates uptake of amino acids, especially serine, arginine, and histidine, and influences serine metabolism and nucleotide synthesis [45], suggesting that intracellular asparagine may play multiple important roles in maintaining cell amino acid homeostasis and cancer cell proliferations.

It has been reported that cancer cells with high NRF2 activation due to KEAP1/NRF2 mutation or KRAS activation depend upon serine and asparagine synthesis pathways for rapid proliferation and survival [39, 40, 46]. Conversely, inhibiting those metabolic pathways or depleting specific amino acids, such as L-asparagine, is toxic to cancers cells that rely on serine or asparagine synthesis [39, 40, 47–50]. In the present study, NSLC01 dramatically inhibited the biosynthesis of multiple amino acids, including asparagine, in NSLC01-sensitive cells. Our data further support that NSLC01 inhibits translation of the key proteins involved in asparagine biosynthesis by inhibiting the cascade of protein translation elongation and upregulating the inhibitory phosphorylation of eEF2. But the de novo syntheses of other amino acids were not affected possibly because de novo synthesis is not the major metabolism pathway for those amino acids or that their synthesis enzymes have slow protein turnover. However, more work is needed in this regard to further validate such hypothesis. A recent study indicates that NRF2 modulates mRNA translation, particularly, the translation of EGFR, to promote pancreatic cancer survival through a reduction in cellular ROS levels which maintains activation of translational regulatory proteins [16]. Whether NRF2 affects eEF2K phosphorylation levels in pancreatic cancer cells remains to be determined. Together, these observations and our data provide a rationale for targeting protein translation pathways in highly NRF2-activated cancers.

In addition to translation initiation, translation elongation also plays a crucial role in cancer survival mediated by oncogenic signaling [51]. The key step in translation elongation control is regulated by eEF2K, which inhibits ribosomal translocation along the mRNA through phosphorylating and inhibiting eEF2 [52]. In our study, NSLC01 selectively activated eEF2K by inhibiting its phosphorylation at S366. Activated eEF2K phosphorylates the downstream eEF2 at T56, thereby inhibiting its translation elongation function. The result, ultimately, is inhibition of the translation of ASNS, GHPDH, PSAT1, and PSPH, key molecules in asparagine and serine synthesis. However, the role of eEF2K in cancer is context-dependent; in some cancers types, the increased eEF2K activity has been shown to inhibit translation, allowing cancer cells to adapt to stressful conditions [53]. More studies are necessary to explore the mechanism of selective translation inhibition by NSLC01 in NRF2-activated pancreatic cancers.

In summary, we have identified NSC84167 as an NRF2 synthetic lethal agent, a potential candidate for targeting NRF2-activated pancreatic cancers. Extensive drug development studies will be necessary to explore its direct targets and pharmacokinetics, enhance selective anticancer activity through development of optimized analogs, establish appropriate formulation methods, and test it in combination with other existing therapies. Given that NRF2 activation occurs in a large subset (~93%) of pancreatic cancers, further optimization and formulation of this lead compound may provide a novel biomarker-guided therapeutic approach to treating pancreatic cancer.

## Supplementary information

Supplementary Figure legends.

Supplementary Fig. 1.

Supplementary Fig. 2.

Supplementary Fig. 3.

Supplementary Tables.
