# Supplementary material for: Compound NSC84167 selectively targets NRF2-activated pancreatic cancer by inhibiting asparagine synthesis pathway
Source: Cell Death Dis. 2021 Jul 10;12(7):693. doi: 10.1038/s41419-021-03970-8 (PMC8272721; doi:10.1038/s41419-021-03970-8)
Supplement: Supplementary file 1 — Supplementary Figure legends. [file 41419_2021_3970_MOESM1_ESM.docx]

**Supplementary figure legends**

Supplementary Figure 1，The effects of NSCL01 on normal pancreatic ductal epithelia cell and non-small cell lung cancer cell lines with or without *KEAP1* gene mutation. A, cells were treated with different doses of NSLC01 for 72 hours, and cell viabilities were assessed with MTT method. B, mutation status of *KEAP1* genes in non-small cell lung cancer cells lines.

Supplementary figure 2, Real-time PCR analysis of RNA expression levels of PHGDH, PSAT1, PHSH, and ASNS in resistant and sensitive cells after treatment with NSLC01 for 24 hours.

Supplementary Figure 3，The effects addition of exogenous asparagine on cell growth inhibition induced by NSCL01. A and B, Cell were treated with different doses of NSLC01 in cell culture medium supplemented with extra asparagine (0.5 mM) or PBS control for 72 hours, and cell viabilities were assessed with MTT assay (*P<0.05; data are presented as mean ± SD., n=3).
