# Supplementary figures and images for: Compound NSC84167 selectively targets NRF2-activated pancreatic cancer by inhibiting asparagine synthesis pathway
Source: Cell Death Dis. 2021 Jul 10;12(7):693. doi: 10.1038/s41419-021-03970-8 (PMC8272721; doi:10.1038/s41419-021-03970-8)

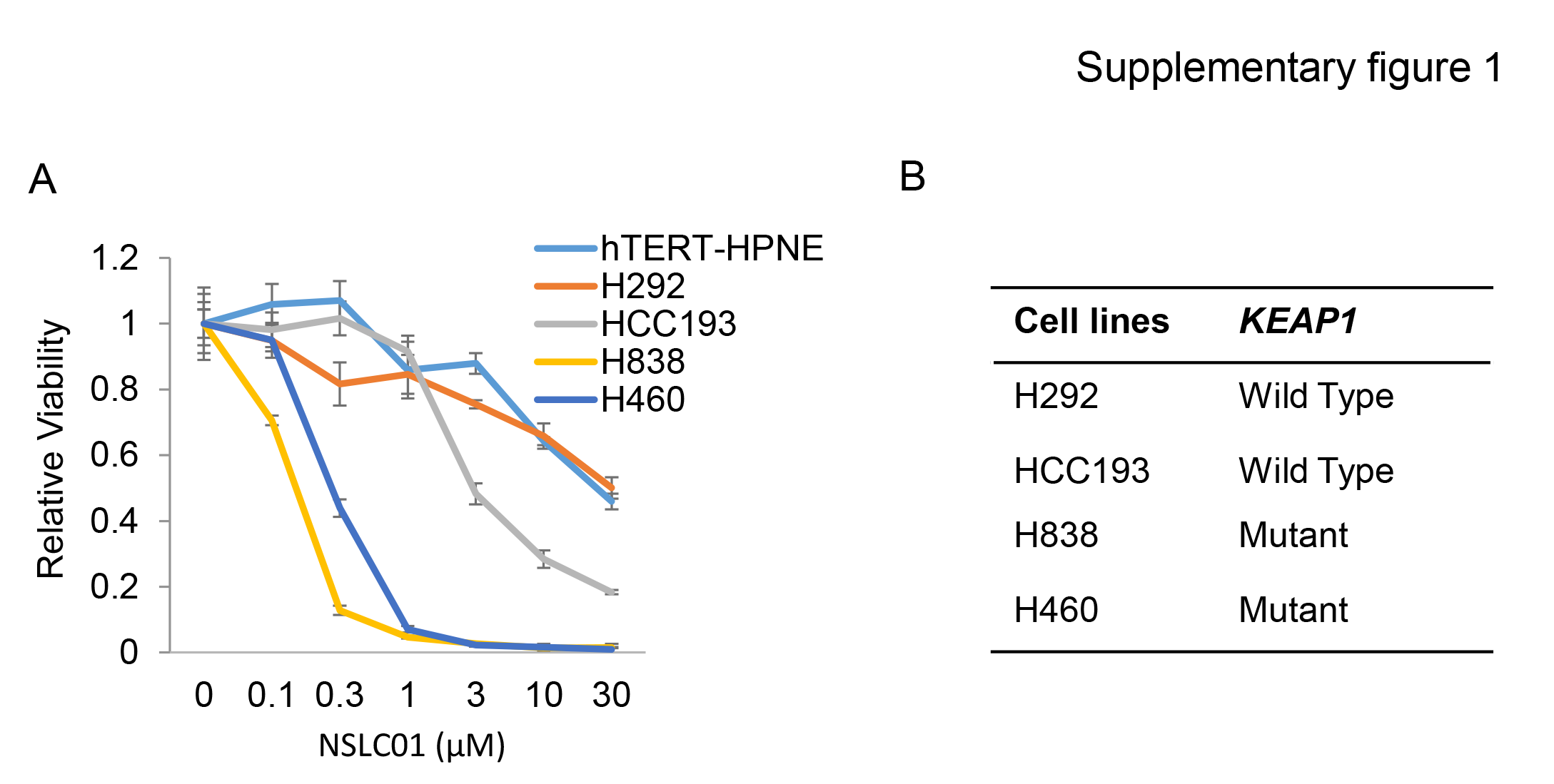

Supplement: Supplementary file 2 — Supplementary Fig. 1. [file 41419_2021_3970_MOESM2_ESM.tif]

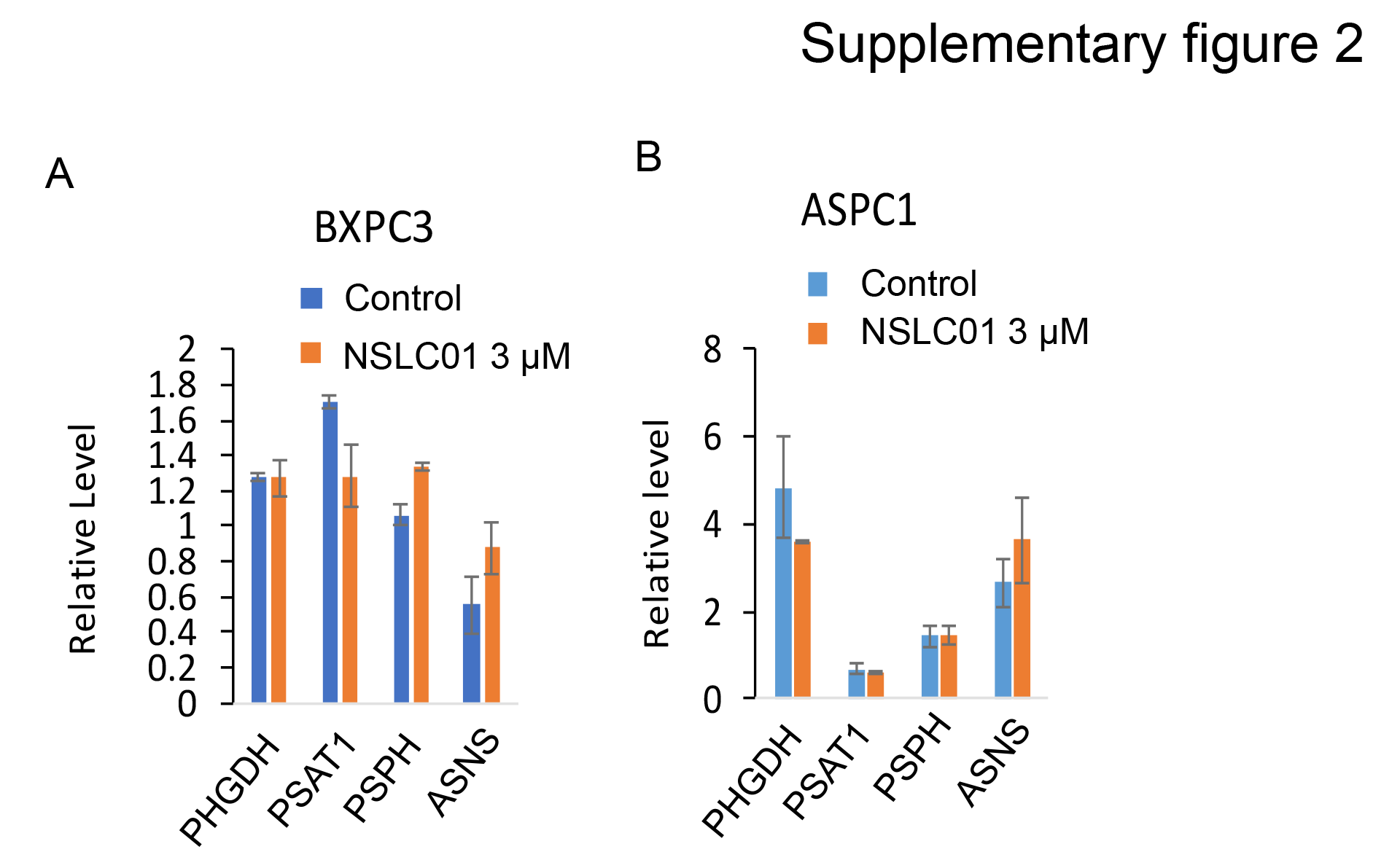

Supplement: Supplementary file 3 — Supplementary Fig. 2. [file 41419_2021_3970_MOESM3_ESM.tif]

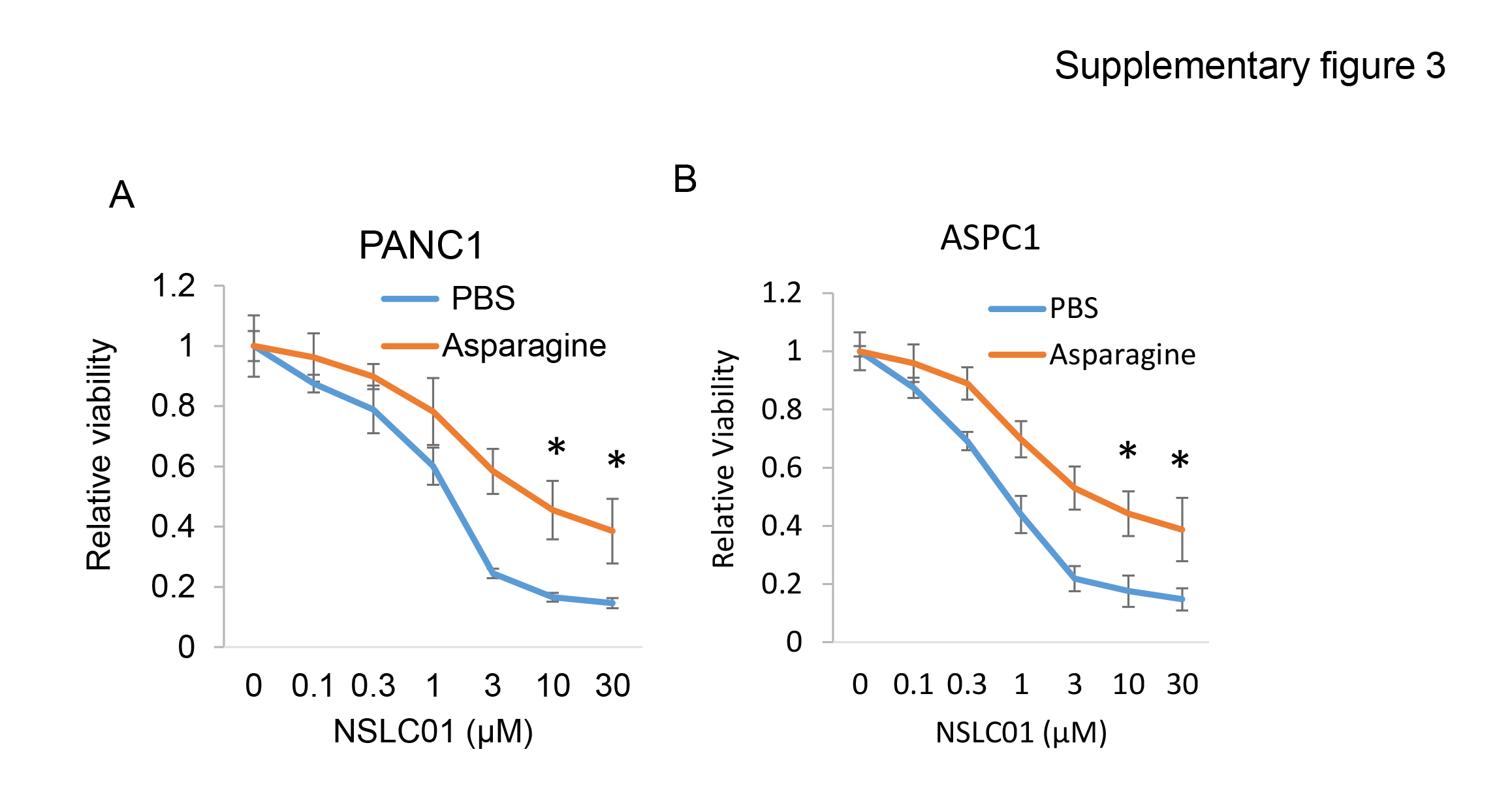

Supplement: Supplementary file 4 — Supplementary Fig. 3. [file 41419_2021_3970_MOESM4_ESM.tif]
