# Supplementary material for: Compound NSC84167 selectively targets NRF2-activated pancreatic cancer by inhibiting asparagine synthesis pathway
Source: Cell Death Dis. 2021 Jul 10;12(7):693. doi: 10.1038/s41419-021-03970-8 (PMC8272721; doi:10.1038/s41419-021-03970-8)
Supplement: Supplementary file 5 — Supplementary Tables. [file 41419_2021_3970_MOESM5_ESM.docx]

**Supplementary Table 1.** Top compounds that correlated with NQO1 expression in the NCI-60 cell line database.

| **NSC number** | **r** | ***P* value** |
| --- | --- | --- |
| 2785 | 0.785 | 0 |
| 84167 | 0.71 | 0 |
| 668896 | 0.68 | 0 |
| 753790 | 0.65 | 0 |
| 658257 | 0.65 | 0 |
| 713198 | 0.62 | 0 |
| 651086 | 0.61 | 0.000002 |
| 624806 | 0.56 | 0.000004 |
| 750104 | 0.56 | 0.000004 |
| 647939 | 0.57 | 0.000005 |

| **NSC number ^n^** | **Correlations r** | **P-value** |
| --- | --- | --- |
| 84167 | 0.498 | 0.00006 |
| 627727 | 0.545 | 0.00009 |
| 671401 | 0.497 | 0.000099 |
| 2785 | 0.483 | 0.000142 |
| 735780 | 0.482 | 0.000149 |
| 762986 | 0.471 | 0.000246 |
| 636869 | 0.508 | 0.000309 |
| 753790 | 0.447 | 0.00039 |
| 712237 | 0.452 | 0.000419 |
| 667078 | 0.468 | 0.000467 |

**Supplementary table-2:** Correlation between expression level of HO-1 gene and drug activities in NCI-60 database

**Supplementary table-3:** Mutations of selected genes in PATC lines

| PATC | KRAS | TP53 | ATR | BRCA1 | BRCA2 | ATM | GNAS | PALB2 |
| --- | --- | --- | --- | --- | --- | --- | --- | --- |
| PATC107 | Mut | Mut | Mut |  |  |  | Mut |  |
| PATC102 | Mut | Mut | Mut |  | Mut |  |  |  |
| PATC153 | Wild | Mut |  |  |  |  |  |  |
| PATC148 | Mut | Mut |  |  |  |  |  |  |
| PATC53 | Mut | Mut | Mut |  | Mut |  |  |  |
| PATC50 | Mut | Mut | Mut |  |  |  | Mut | Mut |
| PATC43 | Mut | Mut |  |  |  |  | Mut |  |
| PATC108 | Mut | Mut |  |  | Mut |  | Mut |  |
| PATC124 | Mut | Mut | Mut | Mut |  | Mut |  |  |
